# Preconditioning with Short-Term Dietary Restriction Attenuates Cardiac Oxidative Stress and Hypertrophy Induced by Chronic Pressure Overload

**DOI:** 10.3390/nu13030737

**Published:** 2021-02-26

**Authors:** Miyuki Kobara, Nessa Naseratun, Hiroe Toba, Tetsuo Nakata

**Affiliations:** Department of Clinical Pharmacology, Division of Pathological Science, Kyoto Pharmaceutical University, Kyoto 607-8414, Japan; nasera0015@gmail.com (N.N.); toba@mb.kyoto-phu.ac.jp (H.T.); nakata@mb.kyoto-phu.ac.jp (T.N.)

**Keywords:** dietary restriction, cardiac hypertrophy, oxidative stress, mitochondria, apoptosis

## Abstract

Left ventricular (LV) hypertrophy and associated heart failure are becoming a more prevalent and critical public health issue with the aging of society, and are exacerbated by reactive oxygen species (ROS). Dietary restriction (DR) markedly inhibits senescent changes; however, prolonged DR is difficult. We herein investigated whether preconditioning with short-term DR attenuates chronic pressure overload-induced cardiac hypertrophy and associated oxidative stress. Male c57BL6 mice were randomly divided into an *ad libitum* (AL) diet or 40% restricted diet (DR preconditioning, DRPC) group for 2 weeks prior to ascending aortic constriction (AAC), and all mice were fed *ad libitum* after AAC surgery. Two weeks after surgery, pressure overload by AAC increased LV wall thickness in association with LV diastolic dysfunction and promoted myocyte hypertrophy and cardiac fibrosis in the AL+AAC group. Oxidative stress in cardiac tissue and mitochondria also increased in the AL+AAC group in association with increments in cardiac NADPH oxidase-derived and mitochondrial ROS production. LV hypertrophy and associated cardiac dysfunction and oxidative stress were significantly attenuated in the DRPC+AAC group. Moreover, less severe mitochondrial oxidative damage in the DRPC+AAC group was associated with the suppression of mitochondrial permeability transition and cardiac apoptosis. These results indicate that chronic pressure overload-induced cardiac hypertrophy in association with cardiac and mitochondrial oxidative damage were attenuated by preconditioning with short-term DR.

## 1. Introduction

Dietary restriction (DR) is defined as a reduced caloric intake without malnutrition and is a powerful non-genomic intervention that has been shown to extend the life spans of experimental animals and prevent age-associated diseases, such as neurodegenerative diseases, cancer, and cardiovascular diseases in experimental animals and humans [1,2,3]. DR was found to prevent major risk factors for cardiovascular diseases, such as hypertension, dyslipidemia, and glucose intolerance, in experimental animals and humans [1,2,3,4]. It also protected against aging- and diabetes-related cardiomyopathy [5,6], ischemic heart diseases [7], and cardiac hypertrophy [8,9] in animal models. Previous studies also demonstrated that DR attenuated diastolic dysfunction in experimental animals and humans [6,10]. Extensive research on DR indicated that the mechanisms underlying its beneficial effects involve the suppression of insulin/insulin growth factor signaling, the target of rapamycin (TOR) pathway, reactive oxygen species (ROS) production, the promotion of the 5′adenosine monophosphate-activated protein kinase (AMPK), and sirtuin signaling pathways [2,11,12,13,14]. Among the various DR protocols proposed to date and examined in animal and human studies, life-long DR is severe and impractical in humans, and, thus, its clinical relevance is limited [15]. Alternative DR protocols, such as intermittent fasting and fasting-refeeding, have been investigated and shown to increase life span and reduce age-related cardiovascular risks [16,17]. Accumulating evidence suggests that the beneficial effects of DR rapidly occur upon its initiation [18,19]. A previous study showed that only 1 week of DR achieved ischemic tolerance in mouse models [19].

Left ventricular (LV) hypertrophy has been identified as one of the independent risk factors for chronic heart failure and sudden cardiac death [20]. The progression of left ventricular hypertrophy and impairments in LV diastolic function occur with aging [21], and the number of patients with heart failure and heart failure-related hospitalizations has been increasing; therefore, it is emerging as a public health issue in developed countries [22]. ROS and oxidative stress play important roles in cardiac hypertrophy and associated heart failure [23,24,25,26]. In isolated myocytes, increases in ROS have been shown to contribute to hypertrophy induced by well-known stimuli, such as angiotensin II and tumor necrosis factor α [24], and these increases also promoted the proliferation of fibroblasts and interstitial fibrosis [27]. Sources of ROS production include NADPH oxidase, mitochondria, uncoupled endothelial nitric oxide synthase, and xanthine oxidase [26,28,29,30], with NADPH oxidase and mitochondria playing crucial roles in pressure overload-induced cardiac hypertrophy and heart failure [26,31]. Therefore, the present study investigated the preconditioning effects of short-term DR on pressure overload-induced cardiac hypertrophy and associated oxidative stress in mice.

## 2. Materials and Methods

### 2.1. Animals and Experimental Protocols

All procedures conformed to the Guide for the Care and Use of Laboratory Animals published by the US National Institutes of Health and the rules of the Declaration of Helsinki. The study protocol was approved by the Bioethics Committee of Kyoto Pharmaceutical University (permission number: CPCO-20-002) and was conducted in accordance with the rules for Animal Experimentation of Kyoto Pharmaceutical University.

**Figure 1:**
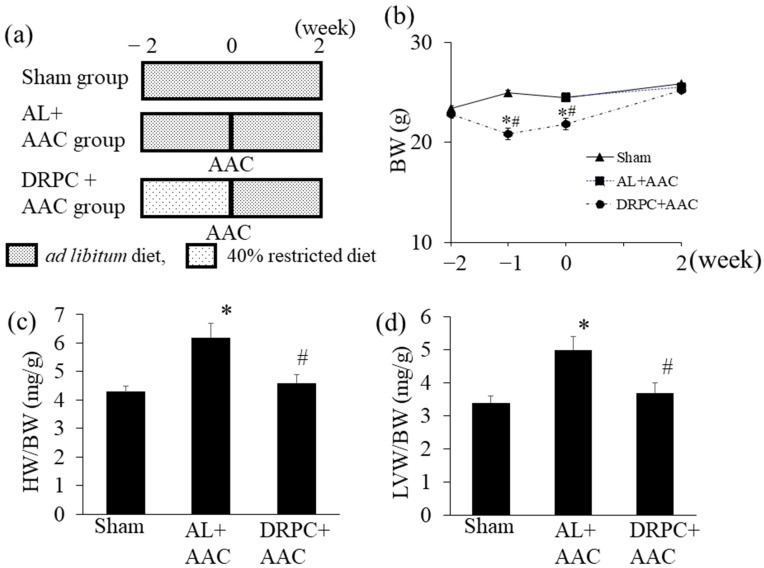
Experimental protocol and effects of dietary restriction preconditioning on body and heart weights. (**a**) Schematic of the experimental protocol adopted for the Sham (*ad libitum* diet without ascending aortic constriction), AL+AAC (*ad libitum* diet with ascending aortic constriction), and DRPC+AAC (preconditioning with 40% dietary restriction followed by ascending aortic constriction under the *ad libitum* diet) groups. (**b**) Time course of changes in body weight during the experimental period (Sham: *n* = 9, AL+AAC: *n* = 8, DRPC+AAC: *n* = 9). Ratios of heart weight to body weight (**c**) and left ventricular weight to body weight (**d**) 2 weeks after surgery (Sham: *n* = 4, AL+AAC: *n* = 7, DRPC+AAC: *n* = 4)). * *p* < 0.05 versus Sham, # *p* < 0.05 versus AL+AAC. HW, heart weight; BW, body weight; LVW, left ventricular weight.

Male c57BL6 mice (6 weeks of age) were obtained from Japan SLC Inc. (Hamamatsu, Japan), housed in individual cages, and fed a standard rodent diet *ad libitum* for 2 weeks. The standard rodent diet (359 kcal, 100 g) comprised the following: 7.9 g water, 23.1 g crude protein, 5.1 g crude fat, 2.8 g crude matter, 2.8 g crude fiber, and 55.3 g nitrogen-free extract. Average dietary intake was calculated from daily food intakes for 2 weeks. Mice (8 weeks of age) were randomly divided into 3 groups: (1) and ascending aortic constriction (AAC) with an *ad libitum* intake (AL+AAC) group, (2) AAC with DR preconditioning (DRPC+AAC) group, and (3) an *ad libitum* intake without AAC (Sham) group. Mice in the AL+AAC and Sham groups were fed *ad libitum*, whereas mice in the DRPC+AAC group were fed 60% of the average caloric intake for 2 weeks. Restricted diets were enriched with vitamins and minerals to avoid malnutrition. At 10 weeks of age, AL+AAC and DRPC+AAC mice were anesthetized by the inhalation of isoflurane. Following tracheal intubation and under controlled ventilation, pressure overload was produced by AAC as previously described [12]. The Sham group was treated similarly, except that the suture around the ascending aorta (AA) was not tied. After surgery, all mice were fed the standard diet *ad libitum* for 2 weeks. A treatment summary is shown in Figure 1a.

### 2.2. Sample Collection and Histological Analysis

Before and 2 weeks after surgery, mice were sacrificed and excised hearts were weighed. The LV was separated from the right ventricle, weighed, and cut transversely at the mid-level. Formalin-fixed paraffin-embedded tissue samples were cut into 4-μm-thick sections and stained with Hematoxylin & Eosin (HE) and Masson’s trichrome. In each mouse that underwent AAC, interventricular septum and posterior LV wall thicknesses were assessed planimetrically using HE-stained specimens. The size of cardiac myocytes was measured by a cross-sectional area assessment using HE-stained sections. To measure cross-sectional myocyte areas, a suitable area of the section was defined as one with circular capillary profiles and myofiber shapes (indicative of a true transverse section). Interstitial and perivascular fibrosis was analyzed in Masson’s trichrome-stained sections. To measure the ratio of interstitial fibrosis, intramuscular blue pixels were counted using a computer-assisted image analysis program, and the ratio of interstitial fibrosis was calculated as the sum of the area of fibrosis divided by the sum of all tissue areas in the field. To assess perivascular fibrosis, blue pixels around intramyocardial coronary arterioles were counted and corrected for the total lumen area. Three to ten independent fields of the myocardium from each rat were photographed using an optical microscope system (Olympus IX71, Olympus Japan, Tokyo, Japan).

### 2.3. Echocardiography

To assess cardiac geometry and function, transthoracic echocardiography was performed 2 weeks after surgery [9]. The left ventricular (LV) end-diastolic diameter (LVDd), LV end-systolic diameter (LVDs), and thicknesses of the interventricular septum (IVST) and left ventricular posterior wall (PWT) were measured using the parasternal short-axis view. The fractional shortening of LV (LVFS) was calculated as LVFS (%) = (LVDd-LVDs)/LVDd × 100. To assess LV diastolic function, the flow velocity of LV filling was assessed by transmitral doppler echocardiography.

### 2.4. Brain Natriuretic Peptide (BNP) mRNA Expression

To assess cardiac BNP mRNA expression, quantitative real-time PCR was performed using the Thermal Cycler Dice Real Time System (Takara Bio Inc, Shiga, Japan) before and 2 weeks after surgery. Total RNA was extracted from LV using ISOGEN II reagent (NIPPON GENE, Tokyo, Japan) and stored at −80 °C. Total RNA was reversed-transcribed using the Takara Reverse Transcription Regent kit (Takara Bio Inc, Shiga, Japan) according to the manufacturer’s instructions. The real-time PCR primer sequences for BNP were forward primer: 5′-GAGGTCACTCCTATCCTCTGG-3′ and reverse primer: 5′-GCCATTTCCTCCGACTTTTCTC-3′. Quantified mRNA expression levels were normalized to that of glyceraldehyde-3-phosphate dehydrogenase (GAPDH).

### 2.5. Immunohistological Staining

The histological expression of 8-hydroxydeoxyguanosine (8OHdG), an indicator of DNA oxidation, and Mac3, a specific antigen of macrophages, was assessed using immunohistological staining. Formalin-fixed paraffin-embedded samples were cut into 4-μm-thick specimens, which were then deparaffinized and placed in reagent containing 3% hydrogen peroxide for 5 min to quench endogenous peroxidase activity. Specimens for macrophage staining were heated in 10 mmol/L citrate buffer (pH6) for antigen retrieval. After blocking non-specific staining with 5% non-fat dry milk and 10% normal goat serum, sections were incubated overnight at 4 °C with an anti-8OHdG monoclonal antibody (1:40 dilution, NIKKEN SEIL Co., Ltd., Shizuoka, Japan) or an anti-Mac3 monoclonal antibody (1:800 dilution, BD Bioscience, CA, USA). Sections for 8OHdG staining were then incubated with a biotinylated goat anti-mouse IgG antibody and streptavidin-peroxidase conjugate, and sections for Mac3 staining were incubated with peroxidase-conjugated anti-rat IgG (Fab’)_2_. This was followed by the addition of 3,3-diaminobenzidine tetrahydrochloride to detect expression and counterstaining with Kernechtrot (for 8OHdG) or hematoxylin (for Mac3). As a negative control, the primary antibody was omitted.

### 2.6. In Situ Detection of Apoptosis

To examine myocyte apoptosis, tissue sections from the middle LV 2 weeks after AAC were stained using the terminal deoxynucleotidyl transferase-mediated dUTP nick end-labeling (TUNEL) method with an APOP TAQ kit (Oncor, Gaithersburg, MD, USA). Formalin-fixed paraffin-embedded samples were cut into 4-μm-thick specimens. After being deparaffinized with xylene, sections were incubated with proteinase K (20 μg/mL) at room temperature for 15 min and treated with 3% hydrogen peroxidase to block internal peroxidase activity. Equilibrium buffer was then applied, and specimens were incubated with terminal deoxynucleotidyl transferase at 37 °C for 1 h. The reaction was stopped with the supplied solution and incubated with anti-digoxigenin peroxidase solution. TUNEL-positive nuclei were visualized with 3, 3′-diaminobenzidine and counter-stained with hematoxylin. As a negative control, terminal deoxynucleotidyl transferase was omitted. We counted 1000 nuclei in each sample under a light microscope and calculated the percentage of TUNEL-positive nuclei.

### 2.7. Mitochondrial Isolation and Measurement of Lipid Peroxide Levels

Heart mitochondria were isolated 2 weeks after surgery. LV tissue was minced into small pieces with scissors and homogenized with a homogenizer (TissueLyser II, QIAGEN, Germany) in mitochondrial isolation buffer (MIB) containing 225 mmol/L mannitol, 75 mmol/L sucrose, and 1 mmol/L EGTA, pH 7.4. The homogenate was centrifuged at 576× *g* at 4 °C for 5 min, and the supernatant obtained was centrifuged at 9000× *g* at 4 °C for 10 min. The pellet was washed in MIB and centrifuged again at 9000× *g*. The mitochondrial pellet was resuspended in MIB buffer. The protein concentration of the mitochondrial suspension was measured using the Lowry method. Mitochondrial oxidative stress was assessed by measuring lipid hydroperoxide levels in mitochondrial suspensions using the Determiner LPO kit (Kyowa Medics Co., Ltd., Tokyo, Japan) according to the manufacturer’s instructions. In brief, the mitochondrial suspension was incubated with pre-treatment buffer (supplied in the kit) at 30 °C for 5 min, coloring buffer (supplied in the kit) was added, and the mixture was then incubated at 30 °C for 10 min. Mitochondrial lipid peroxide levels were measured spectrophotometrically at 675 nm.

### 2.8. NADPH Oxidase-Derived Superoxide Production in Cardiac Tissue

Cardiac NADPH oxidase-derived superoxide production was measured by lucigenin-enhanced chemiluminescence before and 2 weeks after surgery. LV tissue was washed with phosphate-buffered saline (PBS) 3 times to remove blood. Tissue was then minced with scissors and homogenized using the homogenizer in 1 mL of phosphate buffer containing 20 mmol/L potassium phosphate, 1 mmol/L EGTA, 100 mmol/L PMSF, and 1% Proteinase Inhibitor Cocktail (Nacalai Tesque, Inc., Kyoto, Japan), pH 7.0. The homogenate was sonicated using the ultrasonic sonicator and protein concentrations were measured by the Lowry method. Aliquots (30 µL) of the homogenate were incubated in 460 µL of buffer containing 5 µmol/L lucigenin, 50 mmol/L potassium phosphate, 1 mmol/L EGTA, and 150 mmol/L sucrose, pH 7.0. After stabilization, NADPH (100 µmol/L) was added to the sample as a substrate and the chemiluminescence of the sample was measured using a luminometer (Lumat LB9507, Berthold Technologies, Tokyo, Japan) for 5 min. Activity was expressed as relative light units (RLU)/per mg protein/ 5 min.

### 2.9. Measurement of Superoxide Production from Isolated Heart Mitochondria

Mitochondrial superoxide production was measured by chemiluminescence as described previously [31]. Briefly, aliquots of the mitochondrial suspension (described above) were centrifuged at 12,000× *g* at 4 °C for 10 min. The pellet was resuspended to a final mitochondrial protein concentration of 0.05 mg/mL in MOPS buffer containing 20 mmol/L MOPS, 250 mmol/L sucrose, 1 mmol/L EGTA, and 2 mmol/L potassium phosphate, pH 7.4. The mitochondrial suspension (200 µL) and dye L-012 (100 µmol/L, Wako Pure Chemical, Osaka, Japan) were then incubated together. After stabilization, succinate (4 mmol/L final concentration) was added to the sample as the substrate and the chemiluminescence of the sample was measured using the luminometer for 5 min. Activity was expressed as relative light units (RLU)/per mg mitochondrial protein/5 min.

### 2.10. Measurement of Mitochondrial Permeability Transition (MPT)

MPT was assessed by mitochondrial light scattering changes. Briefly, aliquots of the mitochondrial suspension (described above) were centrifuged at 9000× *g* at 4 °C for 10 min. The pellet was resuspended in buffer containing 100 mmol/L KCl, 75 mmol/L mannitol, 25 mmol/L sucrose, 5 mmol/L Tris-phosphate, and 10 mmol/L Tris-HCl, pH 7.4 and centrifuged again at 9000× *g* at 4 °C for 10 min. The pellet was resuspended in assay buffer containing 100 mmol/L KCl, 75 mmol/L mannitol, 25 mmol/L sucrose, 5 mmol/L Tris-phosphate, 10 mmol/L Tris-HCl, 5 mmol/L succinate, and 1 μmol/L rotenone. Light-scattering changes due to swelling were assessed using a spectrophotometer at 540 nm for 10 min. Light scattering changes were expressed as ΔABS/10 min.

### 2.11. Statistical Analysis

**Figure 2:**
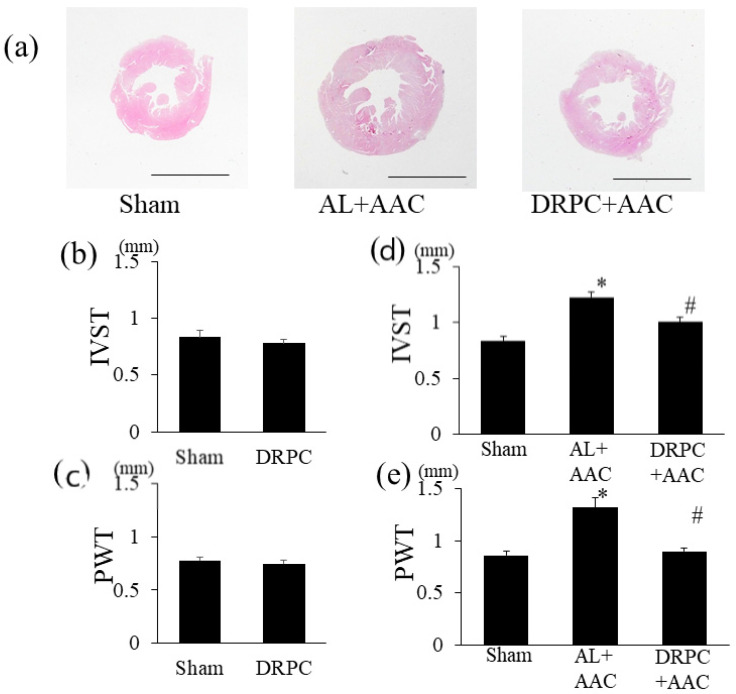
Effects of dietary restriction preconditioning on pressure overload-induced left ventricular wall thickening. (**a**) Representative low-power photomicrographs of hematoxylin & eosin-stained transverse left ventricular sections. Bars indicate 3 mm. Thicknesses of the interventricular septum (IVST) and the posterior wall (PWT) of the left ventricle before (**b,c**) and 2 weeks after surgery (**d,e**) (before surgery, Sham: *n* = 4, DRPC: *n* = 4, after surgery, Sham: *n* = 5, AL+AAC: *n* = 5, DRPC+AAC: *n* = 5). * *p* < 0.01 versus Sham. # *p* < 0.01 versus AL+AAC.

**Figure 3:**
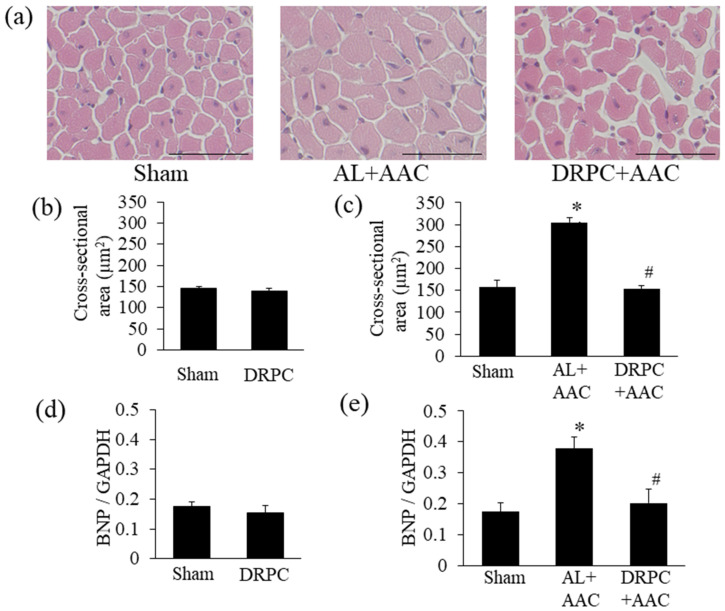
Effects of dietary restriction preconditioning on pressure overload-induced myocyte hypertrophy and BNP mRNA expression. (**a**) Representative photomicrographs of hematoxylin & eosin-stained transverse left ventricular cross-sections 2 weeks after surgery. Bars indicate 50 μm. Cardiac myocyte cross-sectional areas in the left ventricle before (**b**) and 2 weeks after surgery (**c**) (before surgery, Sham: *n* = 5, DRPC: *n* = 7, after surgery, Sham: *n* = 5, AL+AAC: *n* = 7, DRPC+AAC: *n* = 7). BNP mRNA expression in the left ventricle before (**d**) and 2 weeks after surgery (**e**) (before surgery, Sham: *n* = 6, DRPC: *n* = 5, after surgery, Sham: *n* = 6, AL+AAC: *n* = 6, DRPC+AAC: *n* = 5). * *p* < 0.05 versus Sham. # *p* < 0.05 versus AL+AAC.

**Figure 4:**
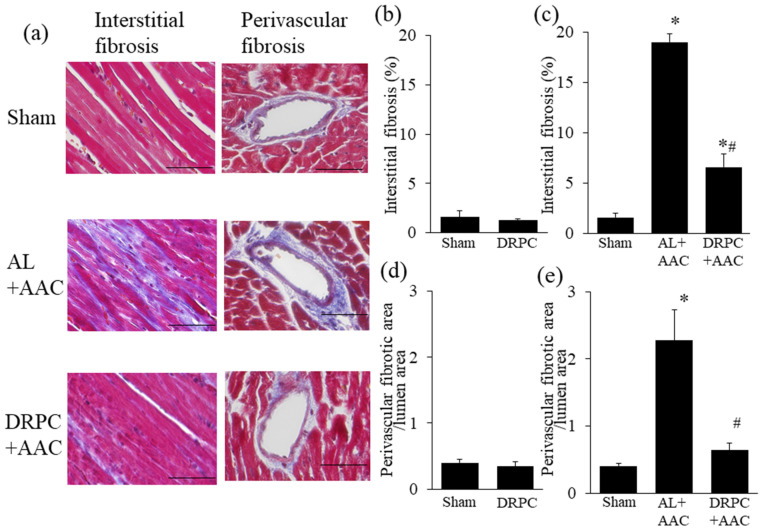
Effects of dietary restriction preconditioning on pressure overload-induced interstitial and perivascular fibrosis in cardiac tissue. (**a**) Representative photomicrographs of Masson’s trichrome-stained left ventricular cross-sections 2 weeks after surgery. Bars indicate 50 μm. Ratio of interstitial fibrosis in the left ventricle before (**b**) and 2 weeks after surgery (**c**) (before surgery, Sham: *n* = 5, DRPC: *n* = 6, after surgery, Sham: *n* = 6, AL+AAC: *n* = 6, DRPC+AAC: *n* = 6). Degree of perivascular fibrosis in the left ventricle before (**d**) and 2 weeks after surgery (**e**) (before surgery, Sham: *n* = 6, DRPC: *n* = 6, after surgery, Sham: *n* = 6, AL+AAC: *n* = 6, DRPC+AAC: *n* = 6). * *p* < 0.01 versus Sham. # *p* < 0.01 versus AL+AAC.

**Figure 5:**
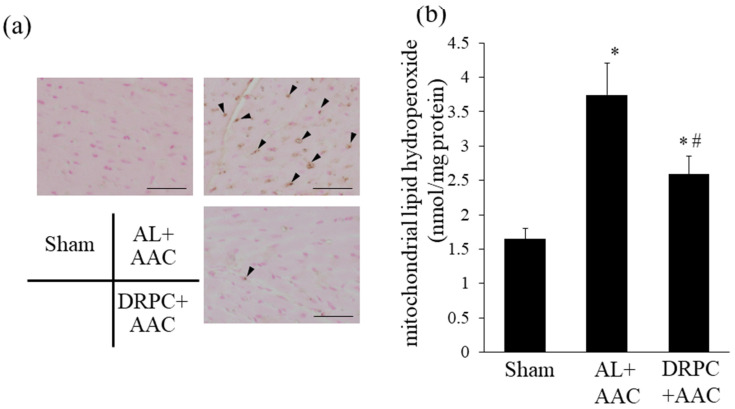
Effects of dietary restriction preconditioning on myocardial and mitochondrial oxidative stress in the left ventricle 2 weeks after surgery. (**a**) Representative photomicrographs of 8-hydroxy deoxyguanosine (8OHdG) immunostaining in the left ventricle. Arrowheads indicate 8OHdG-positive nuclei. Bars indicate 50 μm (*n* = 7). (**b**) Mitochondrial lipid hydroperoxide levels in the left ventricle. Lipid hydroperoxide levels were measured spectrophotometrically, as described in the Methods section (Sham: *n* = 7, AL+AAC: *n* = 6, DRPC+AAC: *n* = 5). * *p* < 0.05 versus Sham. # *p* < 0.05 versus AL+AAC.

**Figure 6:**
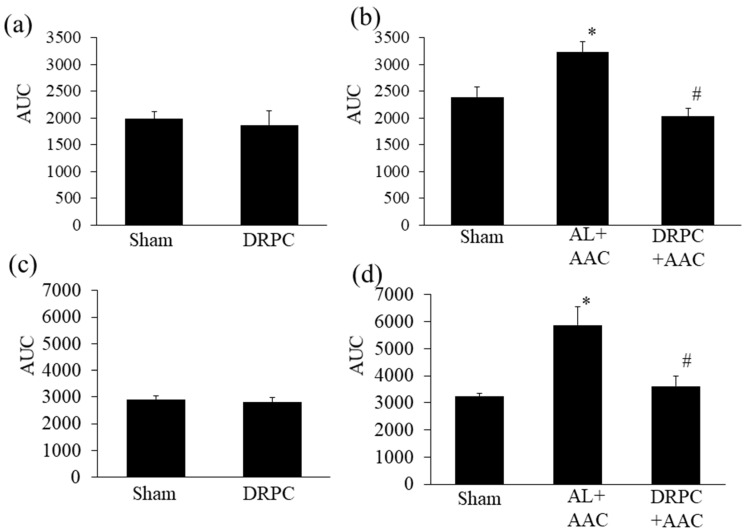
Effects of dietary restriction preconditioning on NADPH oxidase-dependent (**a**,**b**) and mitochondrial (**c**,**d**) superoxide production in LV before (**a**,**c**) and 2 weeks after surgery (**b**,**d**) (before surgery, Sham: *n* = 5, DRPC: *n* = 6, after surgery, Sham: *n* = 6, AL+AAC: *n* = 5, DRPC+AAC: *n* = 6). * *p* < 0.05 versus Sham, # *p* < 0.05 versus AL+AAC.

**Figure 7:**
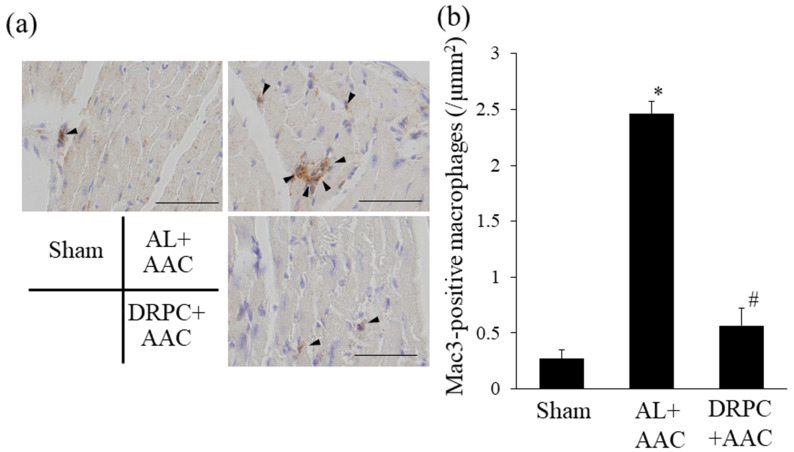
Effects of dietary restriction preconditioning on pressure overload-induced cardiac inflammation. (**a**) Representative photomicrographs of MAC3-positive macrophage infiltration in cardiac tissue 2 weeks after surgery. Arrowheads indicate MAC3-positive macrophages. Bars indicate 50 μm. (**b**) Number of infiltrated macrophages in the left ventricle. (*n* = 3). * *p* < 0.05 versus Sham. # *p* < 0.05 versus AL+ AAC.

**Figure 8:**
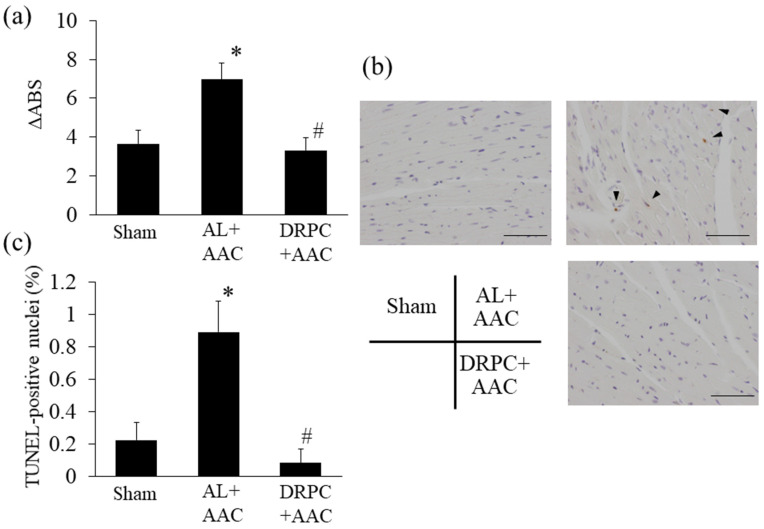
Effects of dietary restriction preconditioning on pressure overload-induced cardiac mitochondrial injury and apoptosis. (**a**) Mitochondrial permeability transition was assessed by light scattering changes 2 weeks after surgery (Sham: *n* = 8, AL+AAC: *n* = 7, DRPC+AAC: *n* = 5). (**b**) Representative photomicrographs of deoxynucleotidyl transferase-mediated dUTP nick end-labeling (TUNEL)-stained LV cross-sections 2 weeks after surgery. Arrowheads indicate TUNEL-positive nuclei. Bars indicate 100 μm. (**c**) Ratio of TUNEL-positive nuclei 2 weeks after surgery (Sham: *n* = 5, AL+AAC: *n* = 5, DRPC+AAC: *n* = 5). * *p* < 0.05 versus Sham. # *p* < 0.05 versus AL+ AAC.

StatView software version 5.0 (SAS Institute Inc., NC, USA) was used to analyze the significance of differences. All values were expressed as the mean ± SEM. Data were analyzed by a one-way ANOVA combined with Fisher’s multiple comparison test (Figure 1b–d, Figure 2c,e, Figure 3c,e, Figure 4c,e, Figure 5b, Figure 6b,d, Figure 7b and Figure 8a,c, Table 1) or the Student’s *t*-test (Figure 2b,d, Figure 3b,d, Figure 4b,d, and Figure 6a,c). A *p* value < 0.05 was considered to be significant.

## 3. Results

### 3.1. Body and Organ Weights

Figure 1b shows the time course of changes in body weight during the experimental period. In the DRPC+AAC group, the initial 2 weeks of DR reduced body weights by 25% of those in the Sham group, and body weights increased after AAC in accordance with *ad libitum* feeding. At the end of the experimental period, body weights were similar among all groups. The ratios of heart weight to body weight and LV weight to body weight were higher in the AL+AAC group than in the Sham group, and significantly decreased in the DRPC+AAC group 2 weeks after surgery (Figure 1c,d). Two weeks of DR markedly decreased body weight, which may have affected the severity of pressure overload induced by AAC. After 2 weeks of DR (just before AAC), no significant differences were observed in the length of the AA circumference with or without DR (AL+AAC: 2.93 ± 0.09 mm, DRPC+AAC: 2.76 ± 0.09 mm). In addition, 3 days after surgery, peak flow verbosity at the constricted ascending aorta was similar in the AL+AAC and DRPC+AAC groups (AL+AAC: 2.73 ± 0.32 m/sec, DRPC+AAC: 2.88 ± 0.24 m/sec).

### 3.2. Assessments of Cardiac Geometry and Function

Cardiac geometry and function 2 weeks after surgery were evaluated by echocardiography (Table 1). IVST and PWT were greater in the AL+AAC group than in the Sham group and significantly less in the DRPC+AAC group 2 weeks after surgery. Systolic function, as represented by LVFS, was similar among all groups. On the other hand, diastolic function, as detected by the E/A ratio, was lower in the AL+AAC group than in the Sham group and was restored in the DRPC+AAC group.

### 3.3. Histomorphometry

Histological LV wall thicknesses in the different groups are shown in Figure 2. Figure 2a shows representative low-power photomicrographs of HE-stained transverse mid-LV sections 2 weeks after surgery. AAC strongly induced symmetric LV hypertrophy in the AL+AAC group, while this was attenuated by DRPC. Preconditioning with 2 weeks of DR did not affect ISWT or PWT before surgery (Figure 2b,d). On the other hand, 2 weeks after surgery, these LV wall thicknesses were markedly increased by AAC and attenuated by DRPC (Figure 2c,e). Myocyte hypertrophy and interstitial and perivascular fibrosis were identified as microscopic changes during the progression of cardiac hypertrophy. Figure 3a and Figure 4a show representative HE- and Masson’s trichrome-stained sections of the LV 2 weeks after surgery. In association with LV hypertrophy, prominent myocyte hypertrophy and interstitial and perivascular fibrosis were observed in the AL+AAC group, and these changes were attenuated by DRPC. The cross-sectional area of myocytes is shown in Figure 3b,c and the area of interstitial and perivascular fibrosis in Figure 4b–e. Preconditioning with 2 weeks of DR did not affect myocyte sizes or the ratio of interstitial and perivascular fibrosis before surgery (Figure 3b and Figure 4b,d). On the other hand, 2 weeks after surgery, myocyte sizes and the areas of interstitial and perivascular fibrosis in the LV increased in the AL+AAC group, and DRPC significantly attenuated the morphological remodeling of LV (Figure 3c and Figure 4c,e).

### 3.4. BNP mRNA Expression in the Hypertrophic Myocardium

The expression of BNP mRNA in cardiac tissue, an indicator of myocyte hypertrophy, before and 2 weeks after surgery is shown in Figure 3d,e. Consistent with the results obtained for the myocyte cross-sectional area, preconditioning with 2 weeks of DR did not affect myocardial BNP mRNA expression levels before surgery (Figure 3d). However, 2 weeks after surgery, the expression level of BNP mRNA was 2-fold higher in the AL+AAC group than in the Sham group, and returned to the Sham-operated level in the DRPC+AAC group (Figure 3e).

### 3.5. Oxidative Stress in the Hypertrophic Myocardium

ROS levels in cardiac tissue 2 weeks after surgery are shown in Figure 5. Figure 5a shows representative staining of 8-OHdG, an indicator of DNA oxidation. AAC increased the number of 8-OHdG-positive nuclei (brown nuclei) in the AL+AAC group, and DRPC attenuated this increase. The mitochondrial content of lipid hydroperoxide, an indicator of mitochondrial oxidative stress, also increased (by up to 2.3-fold) in the AL+AAC group, and DRPC significantly suppressed this increase (Figure 5b).

### 3.6. Myocardial NADPH Oxidase-Dependent and Mitochondrial Superoxide Production

The inhibition of ROS production is an important protective effect induced by DR. Figure 6 shows NADPH oxidase-dependent and mitochondrial superoxide production, major myocardial ROS sources, in the LV myocardium before and 2 weeks after surgery. Preconditioning with 2 weeks of DR did not affect superoxide production derived from either NADPH oxidase or mitochondria (Figure 6a,c). After AAC, NADPH oxidase-dependent and mitochondrial superoxide levels were markedly higher in the AL+AAC group (35 and 80%, respectively) than in the Sham group, and were significantly decreased in the DRPC+AAC group (Figure 6b,d).

### 3.7. Infiltration of Macrophages in the Hypertrophic Myocardium

Increases in oxidative stress exacerbate cardiac inflammation. Therefore, we examined the infiltration of macrophages in cardiac tissue 2 weeks after surgery. Figure 7a shows representative microphotographs of Mac3 staining, a specific marker of macrophages. The number of Mac3-positive macrophages significantly increased in the AL+AAC group, but markedly decreased to the sham-operated level in the DRPC+AAC group (Figure 7b).

### 3.8. MPT and Apoptosis in the Hypertrophic Myocardium

Increases in mitochondrial ROS production and associated oxidative stress induce MPT, leading to apoptosis. MPT was augmented in the AL+AAC group, but markedly decreased to the Sham-operated level in the DRPC+AAC group (Figure 8a). In addition, the number of TUNEL-positive cells, an indicator of apoptosis in cardiac tissue, was increased in the AL+AAC group, but was significantly reduced by DRPC (Figure 8b,c).

## 4. Discussion

The present results demonstrated that preconditioning with short-term DR ameliorated pressure overload-induced cardiac hypertrophy and LV diastolic dysfunction in association with the attenuation of NADPH oxidase-dependent and mitochondrial ROS production and myocardial apoptosis.

In the present study, the increase in LV systolic pressure by AAC was identified as a critical factor promoting LV hypertrophy. During the initial 2 weeks of DR, body and heart weights significantly decreased. In comparisons with the AL+AAC group, a reduction in the aortic transverse diameter and associated changes in the pressure gradient at constriction sites appeared to occur after DR in the DRPC+AAC group. However, the length of the AA circumference just before surgery was similar in DR intake and AL intake mice and the pressure gradient at the constriction site 3 days after surgery was similar between the AL+AAC and DRPC+AAC groups; therefore, the extent of pressure overload appeared to be similar between the AAC groups. DR-induced reductions in blood pressure have been reported in long-term DR animals and obese humans [6,10,32]. However, other animal and human studies demonstrated that DR did not decrease blood pressure [33,34], and we also reported that 4 weeks of 40% caloric restriction in mice did not affect blood pressure [9]. Therefore, the 2 weeks of DR performed in the present study did not appear to affect BP and, as such, the attenuation of LV remodeling by DRPC may be independent of hemodynamic changes.

Oxidative stress plays a critical role in the progression of cardiac hypertrophy by pressure overload [23,25]. In the present study, cardiac oxidative damage was examined by DNA oxidation and mitochondrial lipid peroxidation. The results obtained showed that 2 weeks of pressure overload markedly increased both DNA oxidation and mitochondrial lipid peroxidation, while preconditioning with short-term DR before pressure overload markedly attenuated pressure overload-induced oxidative stress. The DR-induced inhibition of oxidative stress has been reported in a wide range of species and tissues [13,14,35,36]. In rodent cardiac tissue, the beneficial effects of DR on aged and pathological myocardia were mediated by reductions in oxidative stress and ROS-induced damage, as evidenced by the suppression of mitochondrial lipid peroxidation [37] and DNA oxidation [14]. Therefore, the present results are consistent with previous findings. In these studies, DR was continued until oxidative stress assays, while DR in the present model was only continued until pressure overload. Therefore, preconditioning with DR sustained the beneficial suppression of oxidative stress during and following cardiac stress.

In the present study, NADPH oxidase activity and mitochondrial ROS production were investigated to elucidate the mechanisms underlying DR-induced reductions in oxidative stress in cardiac tissue, and the hypertrophy-induced activation of ROS production by both sources was attenuated by DRPC. Accumulating evidence shows that mitochondrial ROS production is attenuated under long- and short-term DR [9,14,38,39] and suppression mechanisms include reductions in oxygen consumption and the membrane potential in mitochondria under DR, thereby inhibiting superoxide production [38,39]. In the present experimental protocol, mitochondria 2 weeks after AAC were not under DR; however, similar mechanisms may have been sustained through the continued beneficial effects of DR. Additionally, the increases in NADPH oxidase activity by pressure overload were attenuated by DRPC. Few studies have compared NADPH oxidase-dependent ROS production under DR with mitochondrial ROS production. Previous studies showed that the expression and activity of NADPH oxidase in the cardiac tissue of a rodent model of metabolic syndrome and pressure overload were both suppressed by DR [6,9]. The mechanisms underlying the suppression of NADPH oxidase activity were not described; however, the inhibition of angiotensin II, a potentiator of NADPH oxidase, by DR has been reported [6] and increases in its levels in cardiac tissue were shown to be involved in cardiac hypertrophy induced by pressure overload [40]. Therefore, the suppression of hypertrophy-induced angiotensin II increases by DR may have decreased NADPH oxidase activity in the present study.

The present results demonstrated that mitochondria from hypertrophic hearts were susceptible to MPT and DRPC suppressed MPT. MPT represents a large conductance pore in the inner mitochondrial membrane, which is closed under non-stressed conditions and sensitized under stress conditions, such as mitochondrial oxidation [30,41]. The induction of MPT reduces the mitochondrial membrane potential, resulting in the release of pro-apoptotic factors from mitochondria [30]. In the present study, preconditioning with DR suppressed MPT and cardiac apoptosis, which is consistent with previous findings [30]. Therefore, the inhibition of mitochondrial ROS production and attenuation of mitochondrial oxidative stress suppressed MPT and apoptosis after DR. A recent study reported that DR activated SIRT3, a mitochondrial deacetylase, and suppressed MPT through the deacetylation of cyclophilin D, a component of the MPT pore [42]. Hence, the activation of SIRT3 as well as inhibition of ROS production may contribute to the preservation of mitochondria.

In the present study, the infiltration of macrophages in cardiac tissue markedly increased in association with myocyte hypertrophy as well as intracellular and perivascular fibrosis in the AL+AAC group. DRPC attenuated pressure overload-induced inflammation and morphological changes in cardiac tissue. Excessive oxidative stress is a well-known inducer of tissue inflammation, including macrophage infiltration, and plays a critical role in myocyte hypertrophy and fibrosis [23,24,25,26,27,43]. In addition, progressive myocyte hypertrophy and cardiac fibrosis also impair cardiac function and continuous pressure overload impairs LV diastolic function before systolic function [26]. The present aortic constriction models showed LV diastolic dysfunction with preserved systolic function. Previous studies demonstrated that antioxidants attenuated pressure overload-induced cardiac hypertrophy and fibrosis [26,40,44] and the genetic deletion of the NADPH oxidase subunit preserved cardiac function after pressure overload in mice [45]. Therefore, the DRPC-induced morphological and functional improvements observed in the present study may be at least partly attributed to antioxidant defenses.

In the present study, preconditioning with short-term DR attenuated pressure overload-induced cardiac hypertrophy. In previous studies on DR-induced cardioprotection, the restricted diet was continued after the stimulation of cardiac injury, such as ischemia-reperfusion and pressure overload [6,9]. We also previously demonstrated that continuous caloric restriction before and after AAC attenuated cardiac hypertrophy [9]. However, in the present study, preconditioning by DR exerted beneficial effects on subsequent cardiac remodeling by pressure overload. Preconditioning with short-term caloric restriction (70% of AL) for only 7 days prior to myocardial infarction in mice was recently shown to reduce infarct sizes and cleaved caspase 3 levels, leading to favorable cardiac function [19]. The present results are consistent with these findings. In addition, the present study revealed that preconditioning with 2 weeks of DR increased tolerance to subsequent pressure overload-induced cardiac oxidative damage for the same period under an *ad libitum* intake. In previous clinical studies on DR, the effects of various DR regimens on age-related diseases, including cardiovascular diseases, and aging-related factors were examined [15,17,34,36,46,47]. The findings of these clinical studies indicated that strict caloric restriction for a long period is beneficial but difficult for humans, and a 30% caloric restriction was not feasible for more than 1 year in the CALERIE-1 study [47]. Hence, feasible diet modifications have been examined, and intermittent fasting was to exert beneficial effects on inflammatory and oxidative stress markers [17,46]. On the other hand, another clinical study reported that a repeated restricted and *ad libitum* diet increased cardiovascular risk factors [48]. Therefore, further investigations are needed to clarify the honeymoon period of DRPC-induced protection and suitable time periods for DR and refeeding. Short-term DR represents a feasible and practical strategy in clinical settings that may precondition cardiac tissue and achieve tolerance to oxidative stress-related cardiac damage, such as LV hypertrophy.

## Figures and Tables

**Table 1:** Echocardiographic data 2 weeks after surgery.

	Sham	AL+AAC	DRPC+AAC
LVDd (mm)	2.58 ± 0.19	2.38 ± 0.12	2.32 ± 0.17
IVST (mm)	0.80 ± 0.03	1.33 ± 0.03 *	0.92 ± 0.06 #
PWT (mm)	0.74 ± 0.04	1.28 ± 0.05 *	0.98 ± 0.04 *#
LVFS (%)	61.5 ± 0.4	67.7 ± 0.3	61.3 ± 0.4
E/A	2.80 ± 0.16	1.64 ± 0.05 *	2.04 ± 0.07 *#

LVDd: left ventricular diastolic diameter, IVST: thickness of the interventricular septum, PWT: thickness of the left ventricular posterior wall, LVFS: left ventricular fractional shortening, E/A: the maximal velocity of early diastole (E wave) to atrial systole (A wave) ratio (Sham: *n* = 5, AL+AAC: *n* = 6, DRPC+AAC: *n* = 5). * *p* < 0.05 versus Sham. # *p* < 0.05 versus AL+AAC.

## Data Availability

The data will be made available from the authors upon reasonable request.
